# Expectation-based types of voluntary sports clubs in regional sports federations

**DOI:** 10.3389/fspor.2023.1200246

**Published:** 2023-06-13

**Authors:** Luc Schulz, Andreas Parensen, Torsten Schlesinger

**Affiliations:** ^1^ Department of Human Locomotion, Chemnitz University of Technology, Chemnitz, Germany; ^2^ Department of Sport Management, Ruhr University Bochum, Bochum, Germany

**Keywords:** service-quality, performance measurement, non-profit management, service provision, governance, professionalization

## Abstract

Sports federations as non-profit organizations play a crucial role for organized sports activities of the population. However, one key responsibility of sports federations is providing needs-based support services to affiliated member sports clubs. Due to limited resources and simultaneously increasing heterogeneous expectations from their member sports clubs, designing an appropriate service portfolio poses increasing difficulties for sports federations. This study addresses these challenges by analyzing member clubs’ expectations and identifying distinct expectation types to enable more individually designed services. To analyze the expectations of member clubs (*n* = 354), the explorative case study was carried out in a regional sports federation in Germany. The findings reveal that member clubs' expectations can be represented using six reliable factors. The subsequent cluster analysis indicates four different expectation-based club types with heterogenous profiles. Based on the z-standardized factor values, the identified club types were labelled as follows: (1) “People Promoters” (32%), (2) “Undemanding” (22%), (3) “Competition-oriented Self-administrators” (23%) and (4) “Demanding Communicators” (23%). The extracted clusters were also reflected and validated by other structural and organizational characteristics of the sports clubs. The extracted types provide a first empirical step to identify different expectation schemes regarding services of sports federations. These schemes enable managers of sports federations to professionalize their service portfolios and, at the same time, to design services contributing to the development of sports clubs in a more targeted manner.

## 1. Introduction

In sports systems in several countries, sports federations play a crucial role in providing organized sports and contribute significantly to fulfilling social policy goals in terms of promoting health and social inclusion (1). In Germany, the federal sport-specific federations, under the umbrella of the German Olympic Sports Confederation, represent the sports federations and the sports clubs at regional and municipal levels (2). This structure corresponds with the federal system in Germany, which is also relevant for the organization of sports. The tasks of the federal sport-specific federations include the development of a particular sport in general, including specific tasks like recruiting, selecting, and supporting talented athletes, organizing sporting competitions, and educating coaches (3). Furthermore, regional sports federations also develop and strengthen the management capacity through consulting voluntary sports clubs (VSC) or provide supportive services for VSCs work and development. Thus, one key responsibility and organizational goal of regional sports federations is the needs-based support of affiliated VSCs as their member organizations through appropriate services. Therefore, the design of services from sports federations should correspond with VSCs interests and expectations (4). However, if the resources collected from member clubs are not sufficient to pursue collective organizational goals of the corporate actor sports federation, the sports federations may be forced to absorb external resources and consequently deviate (in resource allocation) from collective organizational goals (5). This would involve expenditures to incentivize changes in action among affiliated VSCs (5). Due to increasingly scarce resources, and particularly because sports federations must consider demands from multiple stakeholders simultaneously, it is even more important to allocate resources in a targeted manner and to consider the expectations of affiliated VSCs. Designing appropriate services is becoming increasingly challenging for sports federations as interest-driven umbrella organizations due to limited resources and simultaneously increasing heterogeneous expectations from both member clubs and external stakeholders (6). VSCs as members of sports federations show differences in organizational characteristics and contextual backgrounds, which can lead to different challenges (7–9). Therefore, different expectations of VSCs regarding the services offered by their sports federation may exist or arise due to different challenges facing VSCs.

Furthermore, it can be assumed that different expectations lead to differences in the satisfaction of needs and in the perceived quality of services provided by sports federations. Dissatisfaction due to inadequate service quality is usually accompanied by a change of service provider. However, there is a special feature as such an exit strategy is only an option for VSCs with high opportunity costs. Since sports federations generally have a monopoly and sovereignty over sporting competition systems, an exit strategy is linked to the fact that VSCs would also be excluded from established sports competition systems, which would mean a considerable loss of attractiveness for their members. As participation in national and international competitions of the respective sport is only possible within the respective territory of the sports federations, the member organizations are de facto deprived of a realistic “exit option”. While this monopoly ensures clarity in external relations, federation members with differing assessments/opinions in internal relations are, in extreme cases, forced into internal opposition or even “internal emigration” (10, 11). Thus, sports clubs can “voice” (12) their interests and expectations at the federation's general assembly, which seems to be a more functional strategy for VSCs. This means that the clubs articulate their clear expectations for supportive services that sports federations should address. The number of club members is decisive for voting rights, which is capped to limit or even prevent one-sided influence and federation control by larger clubs. The voting rights are exercised by authorized to represent (board) members. Simultaneously, members of the VSCs also can participate in federation committees to pursue their interests, so that there are reciprocal relations between affiliated VSCs and the federation. At the same time, from the perspective of sports federations, it is important to note that if federations are not able to meet the expectations and needs of their member clubs, this can lead to problems in the future development of particular sports, such as membership decline, lack of sporting talents, or financial challenges. In addition, the formation of so-called “wild leagues” can be observed in individual sports (e.g., soccer, darts, International Swimming League), which can undermine the monopoly position of sports federations, if they neglect the expectations of a significant group of member organizations (5). Thus, for the future development of sports federations, it will be of strategic importance that their services are more specifically tailored to the (divergent) expectations and needs of its member clubs. Accordingly, it would be beneficial to gain a specific understanding of what VSCs expect from their sports federation as a service provider in order to optimize their supporting services. However, there are no empirical analyses that reflect what expectations VSCs develop for their sports federation in more detail. To bridge these gaps, this explorative study addresses VSCs' expectations. This leads to research question RQ1: *As members, what are VSCs' expectations of their federation's services, and (ii) how satisfied are they with these services?* Given the heterogeneous expectations, it could have discriminatory potential to classify the VSCs into different groups that could be addressed more individually by sports federations. This leads to research question RQ2: *To what extent can VSCs be classified in terms of expectations regarding their sport federations` services,* and (ii) *how can the identified categories be described in terms of further organizational characteristics*?

## 2. Theoretical approach and literature review

### 2.1. Conceptualization of VSCs expectations

In terms of organizational performance of non-profit organizations (NPOs) like sports federations, researchers have identified specific characteristics that must be considered when measuring performance. Therefore, sports federations need to meet the expectations and needs of their stakeholders with their services (13). The development of performance measurement systems therefore requires that the expectations of the various stakeholders be identified ex-ante in order to determine appropriate performance parameters (14). To address these expectations, performance measurement literature provides a fruitful theoretical frame. There is consensus in the literature that measuring sports federation performance based on stakeholder expectations requires a multidimensional approach. However, there is disagreement about how many and which specific dimensions should be used to measure the performance of sports federations. Different dimensions used in the performance measurement literature are shown in Table 1.

**Table 1:** Dimensions used for performance measurement in sports federations.

Dimensions	Authors
Internal Organization/Function	Daumann et al., 2018 (20); Winand et al., 2010 (13)
Staff & Volunteers	Daumann et al., 2018 (20); Madella et al., 2006 (21)
Leadership & Governance	Daumann et al., 2018 (20); Papadimitriou & Taylor, 2000 (22)
Strategy & Planning	Daumann et al., 2018 (20); Papadimitriou & Taylor, 2000 (22)
Finances	Daumann et al., 2018 (20); Madella et al., 2006 (21); Winand et al., 2010 (13)
Communication	Madella et al., 2006 (21), Winand et al., 2010 (13)
Customers	Winand et al., 2010 (13)
Services	Madella et al., 2006 (21)
Sport	Daumann et al., 2018 (20), Winand et al., 2010 (13)
Athletes	Papadimitriou & Taylor, 2000 (22)
Competition Results	Madella et al., 2006 (21)

Overall, the approaches show some comparable and/or overlapping dimensions that can be grouped into seven distinct core dimensions: (1) Organization and Planning, (2) Personnel, (3) Leadership & Governance, (4) Finances, (5) Communication, (6) Services, and (7) Sport/Talent Development.

Since the approaches consider not only the expectations of the member clubs but also those of external stakeholders, the question arises as to what expectations the member clubs address towards their federation. Precisely because sports federations are interest organizations for which the needs-based support of member clubs is an essential part of their responsibilities (4), the focus of performance measurement should be on aspects important to internal stakeholders. Furthermore, the literature focuses on sports federations at the national level and not at the regional level. Nevertheless, there is some research on member satisfaction in regional sports federations (15), but without using the expectations of the member clubs as a central parameter for organizational performance. Accordingly, this suggests the need for further research that analyzes VSC expectations of their regional sports federation and operationalizes the performance based on the degree to which member clubs’ expectations are fulfilled.

It should be important for VSCs that the federation has clear and transparent management structures. At the same time, the reachability of the federation for individual problems, as well as easy and quick communication of relevant issues are likely to be important for VSCs. Due to the sovereignty over the sporting competition system, it can be expected that especially the organization of competitions and the promotion of young athletes are essential for VSCs. In addition, due to limited resources and competencies of VSCs, support with various consulting services and in personnel development (e.g., coaches, staff) are likely to be important from the VSCs’ perspective.

### 2.2. (Divergent) expectations depending on challenges and characteristics of the VSCs

Expectations of VSCs regarding the services of their sports federation may differ in in relation to the challenges with which VSCs are confronted. Not all services provided by a sports federation may have the same relevance for each VSC. Thus, it is necessary to consider different problems affiliated VSCs face. In the literature, the recruitment and retention of volunteers is identified as a key challenge for sports clubs (7, 9, 16). In addition, problems in recruiting and retaining members (7, 9, 14), coaches (9), and athletes (17) need to be considered when deriving support needs. Furthermore, financial challenges are significant problems for many sports clubs (7, 9). Breuer and Feiler (17) derive immediate support needs from problems and challenges faced by sports clubs. In addition, VSCs need support in designing cooperations, in the field of sports policy and sport facilities, and in the general club organization (17). In reference to underlying tasks and the identified problems and challenges for VSCs, different aspects of supportive services become more or less important. For instance, VSCs that have trouble recruiting new volunteers need more support through personnel management consulting. Other clubs struggle to develop young talents and therefore need support in talent development and/or coach training. VSCs with financial challenges are more likely to need support recruiting and retaining members to generate stable revenue streams and/or financial counseling services. Other VSCs that have more difficulties with cooperations, in the field of sports policy, and/or sport facilities need more support from various advisory services.

The occurrence and severity of VSC challenges depend on organizational backgrounds (7–9). This reveals the need to investigate how VSCs' expectations of their federations differ based on the different challenges posed by different organizational characteristics and contextual backgrounds. VSC expectations can differ both indirectly from challenges resulting from organizational and/or contextual characteristics and directly from organizational and/or contextual conditions.

Researchers have found that organizational characteristics and contextual conditions of VSCs can be related to organizational challenges (7–9, 18). Wicker and Breuer (8) found that VSCs with a larger number of members are more likely to have problems in retaining and recruiting volunteers, and that smaller clubs are more likely to have problems in retaining and attracting athletes and members. Similarly, there is a correlation between the employee structure of VSCs and organizational challenges. VSCs with paid employees tend to have fewer problems in attracting and retaining members than clubs with primarily volunteer staff (18).

Furthermore, Coates et al. (7) state that VSCs problems are related to the financial resources of a club. They show that higher revenues lead to fewer problems in recruiting volunteers. Nevertheless, different sources of income are also relevant for the characteristics of problems in VSCs. Wicker and Breuer (8) state that the amount of income from membership fees influences the retention and recruitment of club members. Thus, clubs with higher income per member have greater problems in this area. Coates et al. (7) found that clubs with sponsorship income face greater financial challenges.

Similarly, the problems and challenges of sports clubs are conditioned by their strategic orientation. Clubs with a clear strategic orientation show fewer organizational problems in financial terms and in recruiting and retaining volunteers (7, 9). In addition, VSCs with a service orientation have fewer financial problems compared to VSCs with a performance orientation (7), and sports clubs with professionalized personnel management have fewer problems in attracting and retaining members and coaches (18). Accordingly, VSCs with more members, which also tend to have paid staff, more professionalized personnel management, and higher revenues through membership fees, can be expected to have fewer expectations of the federation's services in recruiting and retaining members and in retaining and promoting athletes, and vice versa.

In addition to organizational characteristics, however, contextual backgrounds (e.g., geographical area of a club) also have an influence on the nature and manifestation of the challenges faced by VSCs (9). Therefore, it can be expected that VSCs from different regions of a federation have different expectations towards their federation's service portfolio.

## 3. Methods

### 3.1. Contextual background

To analyze expectations of VSCs an (exploratory) empirical case study was carried out in the Bavarian Tennis Federation (BTV), a regional sports federation in the federal state of Bavaria, Germany. The BTV is the largest regional federation within the German Tennis Federation, with 2,260 member clubs (08/2022) and around 300,000 organized tennis players (19). The BTV's 2021 business report shows that the federation had a budget volume of about 5.4 million euros in 2020 (19). Of the total budget volume, the specific source of income of EUR 4.2 million is publicly available. Around 60% of the publicly available income (EUR 4.2 million) was attributable to membership fees (the affiliated VSCs pay membership fees based on the number of their members and teams), which thus accounts for the largest share. About 25.5% of the publicly available income was from government funds and grants, about 14.5% was due to asset management, and less than 0.3% was due to special purpose operations. In 2019, the BTV had a budget volume of about 6.2 million euros and the share of membership fees of the publicly available revenue accounted for about 75.7% (19). This shows that both the budget volume and the share of membership fees decreased and accordingly a more targeted allocation of resources and a greater consideration of the interests of the paying member clubs are of increasing importance for the BTV. In return, the BTV offers its member clubs various services, like organizing competitions mainly in Bavaria, licensing coaches, and promoting young athletes. In addition, the federation acts as a service provider for its VSCs and tries to support them in their daily and strategic work with consulting services (e.g., club development, club management) and needs-based training (e.g., digitalization). In order to position itself more efficiently with regard to the requirements of the federation's work, the BTV has initiated strategic structural reforms to reduce the number of districts and corresponding leagues, to combine fields of responsibilities within regions, and to establish paid management positions in the regions (19).

### 3.2. Data collection and sample

The data were collected from member club representatives in leadership positions. An online survey was conducted to analyze the expectations of member clubs. Using the BTV's email distribution list of all its affiliated VSCs, the federation sent an email to club representatives (e.g., board members, managing directors), which contained a link to the corresponding online survey. This generated a sample of *n* = 354 VSCs (response rate of 17.7%). After data cleaning, a total of 241 responses were available for further data analysis. The sample characteristics show that 57.9% of the clubs surveyed belong to the southern region of the BTV and 42.1% to the northern region. Furthermore, 40.6% of the member clubs were solely tennis clubs and 59.4% were multi-division sports clubs with a tennis division.

Almost half of the clubs in the sample (47.9%) had between 100 and 250 members. 15.8% of the clubs surveyed had fewer than 100 members and 25.4% had between 251 and 500 members. 10.8% of the VSCs in the sample even had more than 500 members.

### 3.3. Measures

Approaches for measuring the performance of sports federations need to include the expectations of member organizations in order to identify appropriate parameters (14). Therefore, the measurement of VSCs expectations was developed based on these approaches [e.g., (13, 20–22)], with separate consideration of the issue of competition organizations as key responsibility of sports federations. The developed item-battery consisted of 57 items that were rated on a 5-point Likert scale showing the levels of importance (from 1 = unimportant to 5 = very important) and satisfaction (from 1 = unsatisfied to 5 = very satisfied).

A significant Bartlett sphericity Test [*x*^2^(903) = 4,738.23; *p* < .01] and the Kaiser-Meyer-Olkin sampling statistic (KMO = .84) showed that the variables were very well suited for an explorative factor analysis (23). After scree plot and further reliability analysis, the exploratory factor analysis with varimax rotation showed the best solution with six reliable factors explaining 60.1% of the variance (Table 2). Only items with factor loading >.50 were retained in the measurement model, except for two items which were retained in the study due to content significance with factor loading of .44 and .49. The six extracted factors with a total of 43 items were labeled as follows: (1) Governance, (2) Reachability and Communication, (3) Youth Talent Development, (4) Support and Consulting Services, (5) Staff Development and Education, and (6) Competition Organization. The analysis of the six factors showed no problems with internal consistency with values for Cronbach's *α* ranging from .77 to .92 (24) and discriminatory power with values of corrected item-total correlation ranging from .39 to .85 (25).

**Table 2:** Statistics for factors of VSCs expectations.

Factors	*M* (SD)	Factor loading	Discriminating power
Governance & leadership (M = 3.68; SD = .74; *α* = .77)			
Communication of decision-making	4.18 (0.99)	.67	.57
Clearly formulated goals and strategies	3.59 (1.12)	.70	.56
Participation opportunities goal setting	3.25 (1.16)	.53	.65
Transparent organizational structure	3.70 (1.16)	.70	.57
Clearly coordinated competences	3.66 (1.05)	.62	.56
Representing VSCs interests	3.95 (1.11)	.44	.39
Reachability & communication (M = 4.31; SD = .59; *α *= .88)			
Good service quality of the office	4.22 (0.85)	.61	.57
Timely response to enquiries from the office	4.42 (0.74)	.71	.67
Reachability of the office	4.08 (0.82)	.56	.53
Friendliness of the office staff	4.12 (0.88)	.69	.65
Competence of the office staff	4.36 (0.77)	.79	.72
Helpfulness of the office staff	4.39 (0.74)	.79	.70
Good internal communication	4.33 (0.75)	.49	.55
A clear homepage	4.35 (0.83)	.55	.53
Current content on the federation's website	4.26 (0.87)	.59	.57
Extensive information on website	4.39 (0.74)	.62	.64
Youth talent development (M = 3.47; SD = .86; *α* = .91)			
Promotion of young athletes	3.53 (1.08)	.73	.70
Clear targets in sporting success	3.13 (1.09)	.68	.66
Competence of trainers	3.52 (1.15)	.82	.77
Accessibility of bases	3.37 (1.11)	.86	.77
Goal-oriented training design	3.67 (1.05)	.75	.74
Coordination between BTV and club trainers	3.72 (1.09)	.73	.68
Competition planning and support	3.35 (1.11)	.66	.69
Regular talent scouts	3.36 (1.15)	.79	.71
Support & consulting services (M = 3.43; SD = .97 a = .92)			
Support in recruiting and retaining members	3.42 (1.17)	.79	.75
Support in club management tasks	3.43 (1.12)	.79	.72
Sufficient club consulting offers	3.30 (1.13)	.87	.85
Sufficient information on club consulting	3.28 (1.07)	.87	.85
Unproblematic agreement of appointments	3.22 (1.18)	.85	.81
A good quality of club consulting	3.74 (1.17)	.74	.71
Competition organization (M = 4.44; SD = .60; *α* = .85)			
Organization of team competitions	4.62 (0.70)	.72	.54
Good planning of the season	4.55 (0.72)	.75	.63
Reasonable reporting deadlines	4.53 (0.70)	.76	.61
An easy-to-use online scorecard system	4.55 (0.77)	.75	.72
An informative online result recording system	4.34 (0.88)	.71	.65
Timely and correctly published results	4.35 (0.88)	.72	.71
An up-to-date and clear ranking list	3.87 (1.12)	.54	.51
Staff development & education (M = 3.53; SD = .82; *α* = .85)			
Cooperations with schools and kindergartens	3.79 (1.03)	.50	.56
Integration	3.29 (1.12)	.76	.72
Inclusion	3.19 (1.12)	.81	.71
Tennis and health	3.73 (1.03)	.70	.65
Development of further club offers	3.46 (1.08)	.63	.62
Digitalization, communication, and new media	3.50 (1.05)	.60	.50

For validation further nominally scaled organizational characteristics relevant to differences in challenges of VSCs [e.g., (7, 9, 16, 17); e.g., region, size] and engagement of the clubs in the federation's communication (e.g., contact frequency, participation in meetings) were examined.

### 3.4. Data analysis

To make initial statements about satisfaction with federation services in the overall group, expectations were evaluated descriptively based on the identified factors by comparing importance and satisfaction [according to (26)].

The six factors were subsequently used to determine expectation profiles of VSCs using cluster analysis. Cluster analysis makes it possible to determine groups based on statistical calculations that are as heterogeneous as possible to each other and homogeneous among themselves (27). First, the hierarchical single linkage method with squared Euclidean distances was conducted. This method forms clusters by merging cases or groups in the iterations based on the smallest distance. This method is therefore particularly suitable for identifying outliers (27). In this process, three outliers were identified and eliminated from the data. A total of *n* = 238 cases (sports clubs) were included in the further cluster analysis.

Subsequently, for the main cluster analysis, Ward's hierarchy method with squared Euclidean distances was carried out using the adapted data set. The hierarchical procedure was chosen because it does not demand the number of clusters a priori. The Euclidean distance measure gives greater weight to larger distance differences and ensures that positive and negative distance differences do not cancel each other out (27). This method forms clusters by merging cases or groups in the iterations in which the increase in variance within a group is as small as possible (27). Since Ward's algorithm generates approximately homogeneous cluster sizes, it is particularly suitable for the classification of balanced expectation profiles. To determine the optimal number of clusters, content considerations were made in addition to the dendrogram and scree plot analysis (27). The stability and quality of the cluster solution was examined regarding interpretability, stability in comparison with a partitioning procedure [k-means method (28)], and heterogeneity between and homogeneity within clusters (27). Z-scores were used to compare the clusters and identify meaningful differences.

To describe and validate the identified clusters further, external validation was performed by analyzing differences between the clusters in relation to organizational characteristics using *x*^2^-tests.

## 4. Results

### 4.1. Expectations and satisfaction of the VSCs

**Figure 1:**
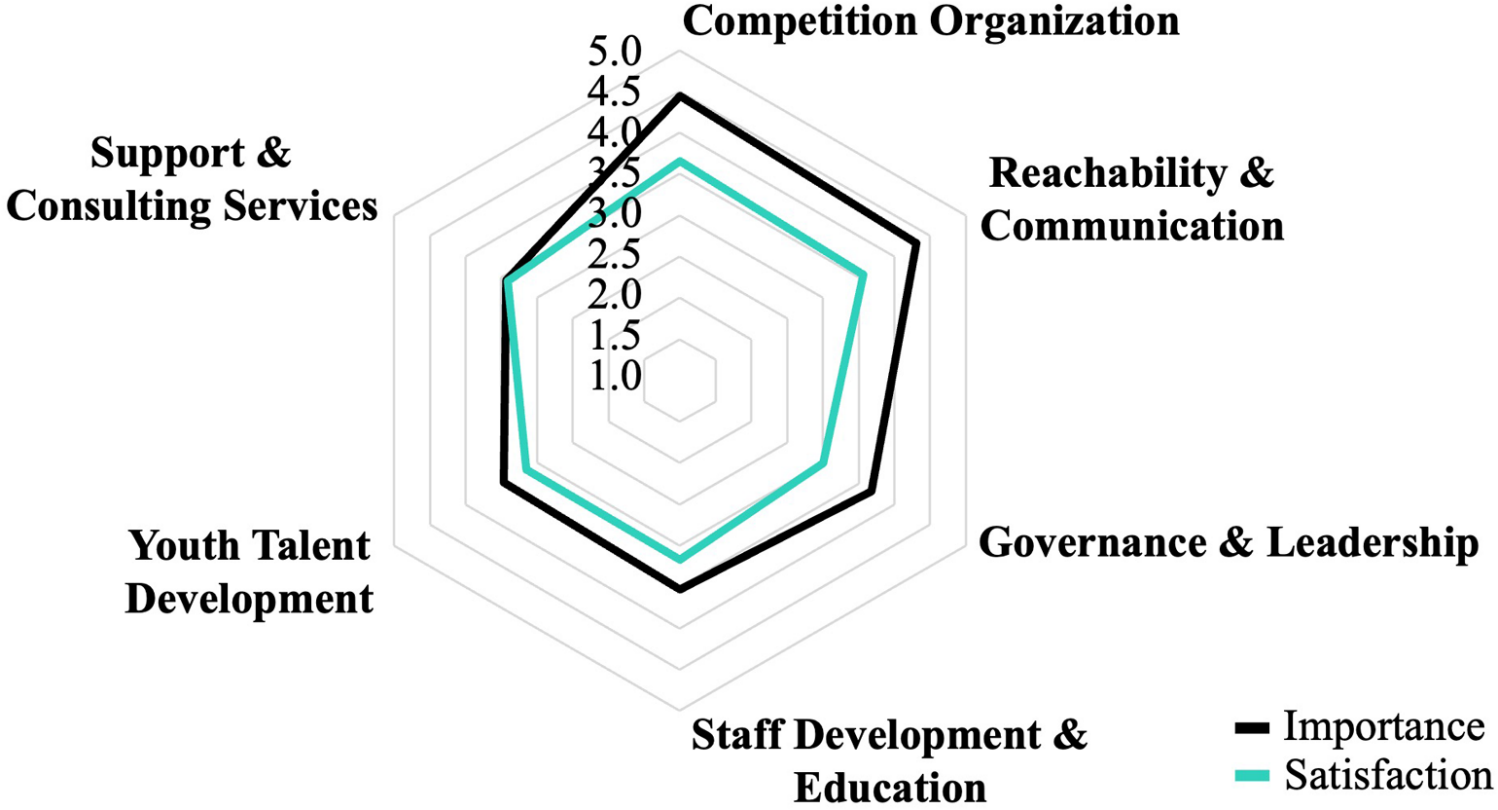
Importance-Satisfaction-Ratio of VSCs according to different dimensions of federation services.

The analysis found that the VSC expectations of their regional sports federation can be represented in six factors. The subsequent descriptive analysis (Figure 1) indicates that the federation faces an overall high level of expectations (*M* > 3.4), particularly for the factors “Competition Organization” (*M* = 4.44; *SD* = .60) and “Reachability & Communication” (*M* = 4.31; *SD* = .59). In addition, the descriptive analysis showed that the VSCs expectations were continually not met. The largest differences were found among the factors with the highest expectations [e.g., “Competition Organization” (Δ = −.78) and “Reachability & Communication” (Δ = −.75)] and vice versa.

### 4.2. Expectation-based profiles of VSCs

**Figure 2:**
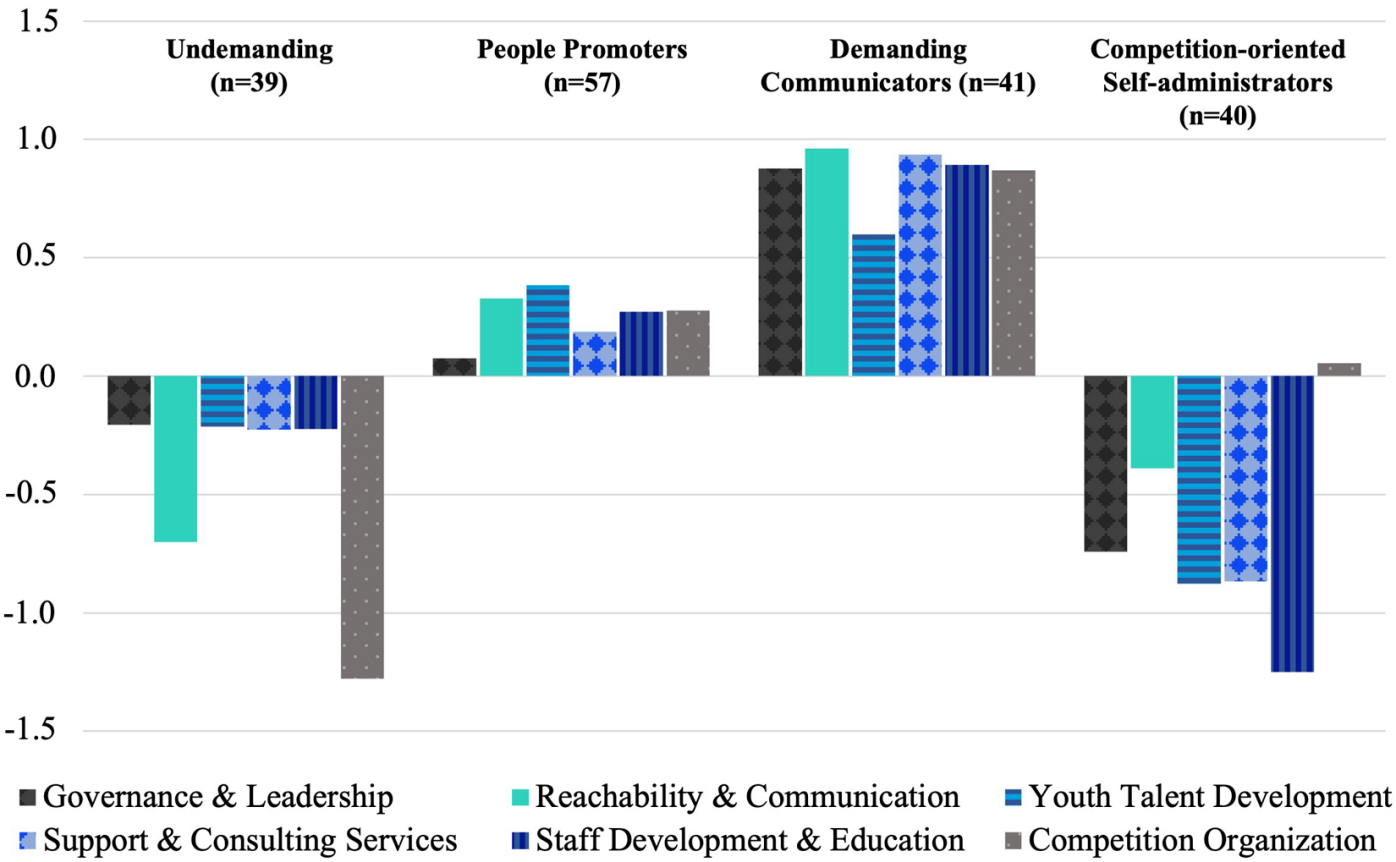
Z-standardized cluster means of expectations in the four types of VSCs.

The cluster analysis showed that the expectations of VSCs have the discriminatory potential to classify the VSCs into homogenous groups (“expectation-related profiles”) that can be addressed more individually by sports federations. An optimal cluster solution was determined using four clusters of relatively similar sizes (Figure 2). Based on the z-standardized factor values, the determined clusters were labeled as follows:

(1) “Undemanding” (*n* = 39, 22%): VSCs in this cluster had below-average expectations across all factors, particularly for the factor “Competition Organization”.
(2) “People Promoters” (*n* = 57, 32%): In addition to “Reachability & Communication”, the VSCs in this cluster had above-average expectations for the factors “Youth Talent Development” and “Staff Development”.
(3) “Demanding Communicators” (*n* = 41, 23%): VSCs in this cluster had higher than average expectations for all six factors, especially for the factor “Reachability and Communication”.
(4) “Competition-oriented Self-administrators” (*n* = 40, 23%): VSCs in this cluster had quiet low expectations, particularly regarding the factors “Governance & Leadership” and “Staff Development and Education”. In contrast, expectations were high for the factor “Competition Organization”.

The k-means cluster analysis showed an acceptable agreement in 68% of the cases, with the final solution based on Ward's method (27). The agreement of the case allocation varied between 87% (Cluster 1) and 42% (Cluster 2). F-values were determined to check for homogeneity. F-values compare the variances within clusters to variances in the overall group. Clusters (1) and (2) were completely homogeneous with F < 1. Only in cluster (3) did the factor “Youth Talent Development” (F = 1.27 > F_crit_ = 1) and in cluster (4) the factor “Governance & Leadership” (F = 1.20 > F_crit_ = 1) reduce the homogeneity. Overall, the homogeneity of the cluster solution was ensured with 22 out of 24 F-values < 1.

### 4.3. Characterization and validation of expectation profiles

To further differentiate the four extracted VSC-types, the clusters were examined in terms of their triggered pressure to act on the federation's service portfolio [according to (29)] and in relation to organizational characteristics. This information contributes to a profound understanding of the four corresponding clusters and enable managers of sports federations to respond to the divergent expectations in a more efficient manner.

**Figure 3:**
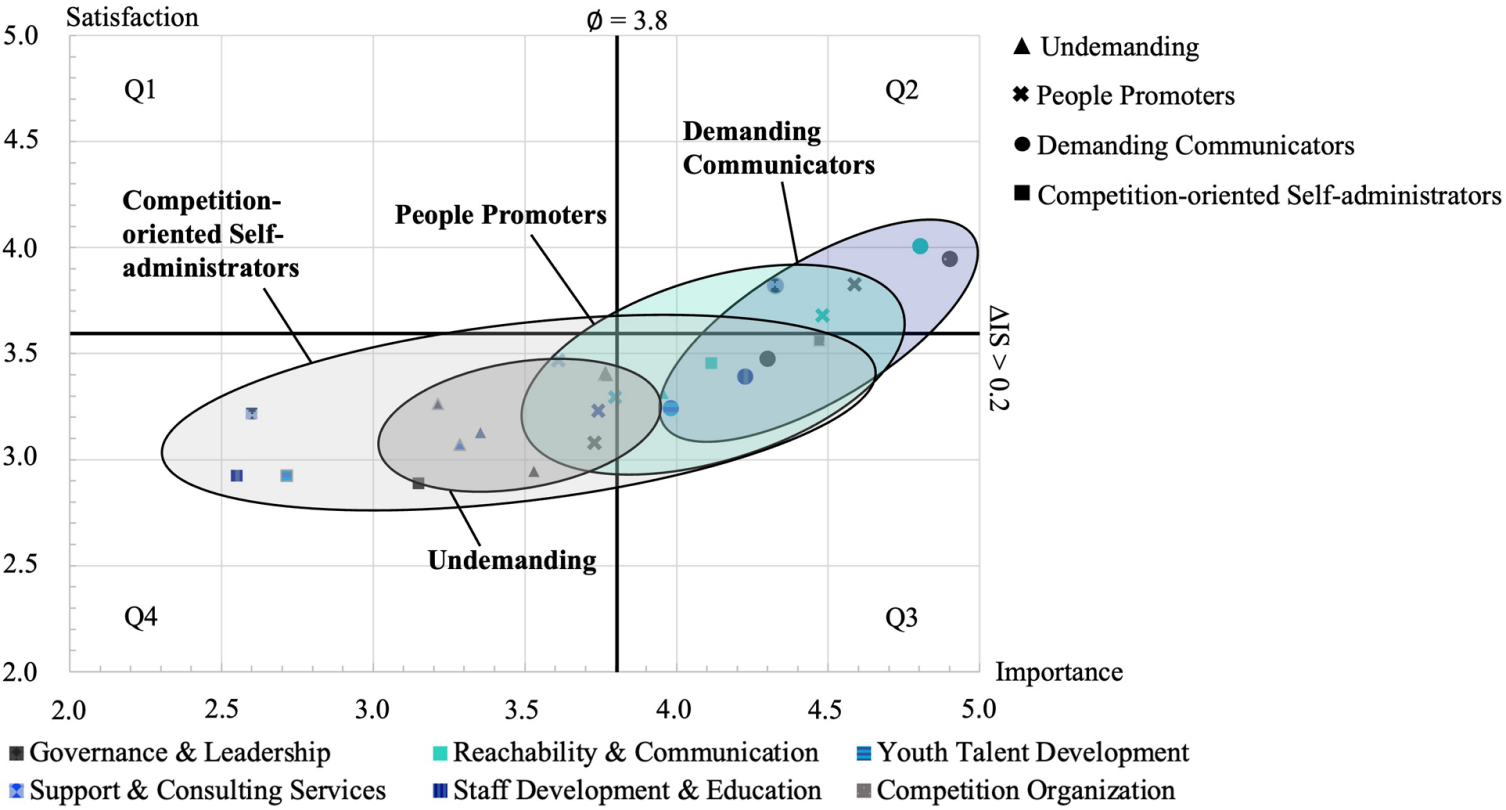
Cluster-specific importance-satisfation-portfolio matrix.

To evaluate triggered pressure to act, both satisfaction values and attached importance values with different expectation dimensions are relevant. These can be graphically displayed in an importance-satisfaction-portfolio [according to (29)] for the four extracted clusters (Figure 3). The expectation-satisfaction-portfolio represents the strengths and weaknesses of the federation's services from the VSC perspective.

The figure shows that the extracted clusters assess the strengths and weaknesses of services differently, and thus trigger different pressures to act for the sports federation. The vertical axis for delineating factors with high importance levels was defined by determining the mean importance value across all factors. Accordingly, any data point that lies above this line has above-average importance (> 3.8). The associated horizontal cut-off was defined by choosing a minimal mean deviation of .2 to obtain a meaningful difference between the importance level and the satisfaction level. In “Q3”, factors with high importance and low corresponding satisfaction levels were grouped together. Accordingly, there is a particular need for the sports federation to adapt specific services. The factors shown in “Q3” can be assigned to the “Demanding Communicators” (50%; (1) “Governance & Leadership”, (2) “Staff Development & Education”, (3) “Youth Talent Development”), the “Competition-oriented Self-administrators” (33%; (1) “Competition Organization”, (2) “Reachability & Communication”), and the “Undemanding” (17%; (1) “Reachability & Communication”). This is plausible insofar as the “Demanding Communicators” expect a high level of service quality in various areas, the “Competition-oriented Self-administrators depend on the federation in terms of “Competition Organization” (sovereignty over sporting competition system), and the “Reachability & Communication” of the federation is critical in everyday business even for largely autonomous clubs. As “Competition Organization” plays a subordinate role for the “Undemanding”, the “Reachability & Communication” of the federation is nevertheless just as critical an aspect for daily club management.

The further analysis of organizational characteristics of the four extracted clusters showed that there are differences in club-related characteristics and in engagement-related characteristics of the VSCs in the federation's communication and development (Table 3).

**Table 3:** Characterization of VSCs based on organizational characteristics.

	Total	CL1	CL2	CL3	CL4	Statistics
Region of Bavaria						
North	41.48	38.46	32.14	46.34	52.50	*χ*^2^ (3,176) = 4.56; *p* = .207
South	58.52	61.54	67.86	53.66	47.50	
Club type						
Single divisional	40.91	48.72	48.21	39.02	25.00	χ^2^ (3,176) = 6.47; *p* = .091
Multi-divisional	59.09	51.79	51.79	60.98	75.00	
Members						
<100	14.20	12.82	17.86	4.88	20.00	χ^2^ (9,176) = 13.49; *p* = .142
100–250	48.86	61.54	37.50	56.10	45.00	
251–500	25.00	17.95	35.71	21.95	20.00	
>500	11.93	7.69	8.93	17.07	15.00	
Large club						
Yes	64.57	55.26	58.93	65.85	80.00	χ^2^ (3,175) = 6.41; *p* = .09
No	35.43	44.74	41.07	34.15	20.00	
Contact frequency (p.a.)						
Never	17.96	37.14	16.36	2.54	18.42	χ^2^ (9,167) = 25.91; *p* < .001***; V = .39
1–3	53.89	34.29	65.45	51.28	57.89	
4–7	16.77	14.29	14.55	23.08	15.79	
>7	11.38	14.29	3.64	23.08	7.89	
Contact medium phone						
Yes	51.98	48.72	45.61	57.50	87.50	χ^2^ (3,177) = 19.32; *p* < .001***; V = .33
No	48.02	51.28	54.39	42.50	12.50	
Participation in BTV meetings (last 10 years)						
Never	16.03	17.65	16.00	8.82	20.00	χ^2^ (9,156) = 16.21; *p* = .063
1–3	30.77	41.18	36.00	29.41	15.00	
4–7	25.64	14.71	28.00	17.65	37.50	
>7	27.56	26.47	20.00	44.12	22.50	
Digital presence						
Yes	25.99	23.08	19.30	41.46	22.50	χ^2^ (3,177) = 6.86; *p* = .076
No	74,01	76.92	80.70	58.54	77.50	

(in %; CL 1 = Undemanding; CL2 = People promoters; CL3 = Demanding communicators; CL 4 = Competition-oriented self-administrators)

*.01 ≤ *p* < .05: significant; **.001 ≤ *p* < .01: very significant; ***p < .001: highly significant.

It is striking that the cluster “Competition-oriented Self-administrators” contains a larger proportion of multi-disciplinary clubs [75%; *χ*^2^(3, 176) = 4.56; *p *= 0.21] and large clubs [80%; defined as >500 members; *χ*^2^(3.175) = 6.41; *p* = 0.09] compared to the total group (59%; 65%). This can be explained by the fact that larger VSCs tend to have paid staff who handle the club management and administration autonomously, and therefore require less support from the federation. However, these differences were not significant. A significant difference was found in the preferred method of contacting the federation [*χ*^2^(3, 177) = 19.32; *p* < .001***; V = 0.33]. 88% of the “Competition-oriented Self-administrators” preferred the phone over other media, whereas in the total group only 52% of the VSCs used the phone. This is presumably due to the fact that paid staff (“Competition-oriented Self-administrators”) are more likely to have the time resources to contact the federation (by phone) during business hours. Another significant difference was observed in the contact frequency of the federation p.a. [*χ*^2^(9, 167) = 25.91: *p* < .001***; V = 0.39]. 23% of the “Demanding Communicators” contacted the federation more than 7 times p.a. (“Competition-oriented Self-administrators” 8%) and only 3% of the “Demanding Communicators” did not contact the federation at all (“Competition-oriented Self-administrators” 18%). At the same time, 44% of the “Demanding Communicators” participated in federation meetings more than 7 times in the last 10 years (“Competition-oriented Self-administrators” 23%) and 9% never participated [“Competition-oriented Self-administrators” 20%; *χ*^2^ (9, 156) = 16.2; *p* = .06]. In addition, it is striking that 41% of the “Demanding Communicators” have a digital presence (> 2 digital channels) in contrast to the total group [26%; *χ*^2^ (3, 177) = 6.86; *p *= .08]. This shows that the “Demanding communicators” do not only expect a high level of service quality but show significant engagement in terms of the federation's development and their own communication and digital presence.

## 5. Discussion

This study investigated VSCs' expectations of their umbrella sports federation at the regional level. First, expectations of VSCs were identified using explorative factor analyses based on concepts for measuring the performance of sports federations. The findings revealed that the addressed expectations of VSCs can be represented in six factors: (1) “Youth Talent Development”, (2) “Reachability & Communication”, (3) “Support & Consulting Services”, (4) “Competition Organization”, (5) “Staff Development & Education” and (6) “Governance & Leadership”. The findings reveal that overall expectations of the federation are high, indicating that sports federations play an important role as service providers for VSCs. Reasons for this might be that VSCs themselves do not have sufficient resources and/or their own administrative service structures to deal with their own problems and challenges. Accordingly, VSCs expect more needs-based support and service orientation from their sports federation. On the other hand, this also reveals that VSCs do not give in to their lack of negotiating power due to limited exit options.

The further analysis of corresponding satisfaction values showed that the high expectations of VSCs regarding their federation's services are continually not met. Therefore, VSCs should articulate their expectations more proactively (“voice”) to improve the service quality of their sport federation, particularly due to their lack of exit options. The greatest differences despite “Competition Organization” were primarily found in business management questions (“Reachability & Communication” and “Governance & Leadership”). This is in line with the literature, which also reveals the need for sports federations to become more “business-like” [e.g., (6, 30, 31)]. This suggests that the federation is also confronted with structural professionalization challenges [e.g., hybrid staff structures (32–34)]. This means that it is not just a matter of the federation addressing specific services, but also of dealing holistically with the increased demands of professionalization.

The VSC classifications showed that there is discriminatory potential in the VSCs' expectations and that there are four VSC types with heterogeneous expectation profiles: (1) “Undemanding”, (2) “People Promoters”, (3) “Demanding Communicators” and (4) “Competition-oriented Self-administrators”. This indicates that not only external stakeholders have different expectations of sports federations (5), but that expectations also differ between member clubs despite their interest-driven umbrella organization. Reasons for this seem to be different problem situations, organizational structures, and/or contextual conditions. This highlights that a classification of VSCs is necessary due to the heterogeneous expectation profiles in order to optimize the service quality of a federation, which is composed of the comparison process between importance and satisfaction [according to (26)].

Accordingly, the VSC expectation-types need to be addressed more individually by sports federations. For this purpose, the clusters were analyzed in terms of their triggered pressure to act on the federation [according to (29)] and in relation to organizational characteristics. Nevertheless, decisions on resource allocation should not be made exclusively based on the pressures of action of the specific club types, but (heterogeneous) demands of external stakeholders should also be considered. For this reason, incomplete satisfaction of the needs of the member clubs may well be rational, because otherwise other stakeholders cannot be taken into account, or only to a lesser extent. Therefore, the federation should act in such a way that the delta between resource investment and expectation fulfillment is as small as possible, while at the same time, the resource expenditure to incentivize behavioral changes of affiliated VSCs should be considered when prioritizing external interests. However, since federations are interest organizations, they cannot entirely neglect specific expectations of clubs. Nevertheless, individual priorities can be set so that expectations of member clubs with high pressure to act (Q3) are prioritized, which ideally also show some congruence with demands from external stakeholders. For example, it seems to be appropriate for the BTV to prioritize the factor “Reachability & Communication”, since this factor is seen as requiring action by two types of clubs (“Undemanding” & “Competition-oriented Self-administrators”; Q3) and there is presumably a high degree of congruence between this factor and the general demands of external stakeholders. At the same time, this factor is the only one with an important need for action from the perspective of the “Undemanding”, for whom there is an increased risk of leaving the federation, since “Competition Organization” plays a subordinate role for these clubs and an exit is therefore associated with lower opportunity costs than for other club types.

Furthermore, it was also found that the extracted clusters are reflected in other characteristics of the organizations. The “Demanding Communicators” in particular consider the federation's service portfolio to be in need of action. At the same time, the “Demanding Communicators” are more involved in the development and communication of the federation and are also more digitally oriented themselves. These findings go hand in hand with the overall high expectation level of the VSCs in this cluster and show up as expected findings. In contrast, “Competition-oriented Self-administrators” are larger clubs that act more autonomously and are less involved in the federation's development and communication. This can be explained by the fact that large VSCs are financially stronger and more professionally positioned (18). Accordingly, large VSCs tend to have sufficient resources and their own more developed administrative service structures, so they have fewer expectations of their federation.

## 6. Limitations and future research

There are some limitations to our explorative case study that must be considered, which at the same time offer perspectives for further research in the field of supportive services in sports federations:

First, the expectations of the VSCs as corporate actors were operationalized based on the assessments by decision makers within the VSCs. Even though this approach is common practice in sports organization research, it can be assumed that the aggregated expectations of individual VSC members (accumulated expectations provided by all club members) may differ from the information provided by the decision makers within the VSCs. This could lead to a distorted picture of expectations when looking at VSCs intra-individually and then comparing them with recorded expectations inter-individually. However, this problem does not only concern the scientific operationalization of VSCs’ expectations but is also reflected in democratic decision-making processes in the context of sports federations. At general meetings of the BTV, for example, only the decision-makers of the VSCs (authorized to represent (board) members) can represent the interests of the VSC and articulate expectations. Second, more in-depth information on the VSCs (e.g., staff structure, strategic orientation, financial resources, professionalization) would contribute to a better understanding of the membership structure and would provide more profound validation opportunities. Third, the data in this case study only refer to one selected regional sports federation in one sport (tennis). The sport tennis is often preferred by people in a better socioeconomic position (35). Scholarly evidence shows that more affluent individuals often participate in sports more and have higher sports expenditures (36, 37). Consequently, it can be assumed that the socioeconomic situation of club members is also reflected in their collective expectations of the federation. In addition, other sports federations may have different financing structures, which are determined less by the member clubs and more by external sources of revenue. As a result, the expectation structures and relationships in the internal and external interactions of the federation would be different. Accordingly, the generalizability of the findings is limited, especially for sports preferred by members with a lower socioeconomic situation [e.g., football (35)] and/or other determinants that influence sports participation (e.g., demographic determinants) that differ from tennis. Therefore, any generalizations of the findings are limited by the regional structure and the specific sport of the study. Future research needs to ask how robust the findings are for other sports and federations with other structural and environmental circumstances. Therefore, replicating studies should test the robustness of the measurement model by confirmatory factor analysis. With a robust measurement model, other federations and confederations can be compared cross-sectionally, and longitudinal comparisons can also be made.

## 7. Conclusion

Overall, it can be said that the extracted VSC types provide a first empirical step to make different expectation schemes of VSCs visible. These schemes enable managers of sports federations to specify their service offer portfolios to respond to the different expectations in the corresponding four clusters in a more targeted and efficient manner. Furthermore, this study contributes to the research of performance measurement of sports federations at the regional level.

## Data Availability

The raw data supporting the conclusions of this article will be made available by the authors, without undue reservation.
